# Research frontiers and hotspots of coronary chronic total occlusion: A bibliometric analysis

**DOI:** 10.1097/MD.0000000000040537

**Published:** 2024-11-15

**Authors:** Shudi Li, Menghe Zhang, Wenwen Li, Zhenhai Sun, Yunxiao Zhang, Yaoyao Zuo, Shouqiang Chen

**Affiliations:** a Shandong University of Traditional Chinese Medicine, Jinan, China; b The Second Affiliated Hospital of Shandong University of Traditional Chinese Medicine, Jinan, China.

**Keywords:** chronic total occlusion, Citespace, coronary artery disease, research frontiers, research hotspots, VOSviewer

## Abstract

By analyzing the relevant literature, we can accurately grasp the current status of diagnosis and treatment of chronic total occlusion of coronary artery, and clarify the development trend, research frontiers and hotspots of this disease. A literature search with “chronic total occlusion” as the title was performed in the Web of Science database. The title, author, abstract, keywords, institution, publication, country, reference, and other endnotes of the selected literature were exported in the form of text. The author, country, institution, and keywords of the literature were analyzed through Citespace and VOSviewer. The United States has the highest proportion of articles. The institution with the largest number of publications in this field is the Minneapolis Heart Institute Foundation. Brilakis Emmanouil S is the author with the most published articles. The journal system with the largest number of articles in this field is Cardiac Cardiovascular Systems. The keywords with the highest frequency are “chronic total occlusion,” “percutaneous coronary intervention,” “recanalization,” and “revascularization.” The burst detection analysis of hot keywords shows that “algorithm,” “management,” and “mortality” are the research hotspots in this field in recent years. At present, the research on this disease mainly focuses on the opening of occluded coronary arteries through various treatment methods. In the near future and the next few years, the research hotspots may be the scoring system algorithms for the treatment of chronic total occlusion of coronary artery and the management strategies for patients.

## 1. Introduction

Chronic total occlusion (CTO) coronary artery disease is defined as the occurrence of complete occlusion of 1 or more coronary arteries, resulting in the interruption of forward blood flow, and the duration of occlusion is >3 months.^[1]^ Coronary CTO lesions is a serious stage of coronary artery disease, and the clinical manifestations are dominated by chest tightness, chest pain, and other manifestations of coronary artery disease. CTO lesions can lead to angina pectoris, myocardial infarction, heart failure, and even sudden death. Epidemiologic investigation shows that the incidence of coronary CTO lesions in patients with conventional angiography is about 20%,^[2]^ accounting for >30% of coronary heart disease, but <10% of patients receive surgical treatment.^[3]^ Currently, there are 3 main treatment modalities for CTO lesions: drug therapy, coronary artery bypass grafting therapy, and percutaneous coronary intervention (PCI).^[4,5]^ Interventional therapy has the advantages of less trauma and faster postoperative recovery, which is the key development direction of current surgical treatment and the most valuable treatment modality for research.^[6–8]^ Studies have shown that recanalization of the coronary artery at occlusion can significantly reduce the incidence of adverse cardiovascular events.^[9,10]^ However, it has been suggested that after the establishment of collateral circulation in the occluded coronary artery, the opening of the occluded coronary artery does not have much effect on the improvement of adverse outcomes in patients.^[11]^ There are still many research gaps regarding the pathogenesis, natural history, and long-term prognosis of CTO lesions after recanalization. As well as the low recanalization rate, high restenosis rate, unclear long-term prognosis, and high risk of intraoperative complications, the treatment difficulties of CTO lesions have hindered the research process of this disease.

The term “bibliometrics” was first coined by Alan Pritchard, a leading British intelligence scientist, in 1969, which enables the use of statistics to illustrate relationships between published studies.^[12,13]^ Citespace and VOSviewer are software used for bibliometric studies.^[14]^ They can extract key information from publications and visualize the dynamic development of a field of research.^[15–18]^ The detection and treatment rates of CTO lesions have been increasing in recent years.^[19]^ For such complex coronary artery disease, it has been the difficulty of clinical treatment and the focus of clinical research,^[20]^ but there is no bibliometric analysis on this disease aspect. In this paper, we visualize and analyze the related literature through Citespace and VOSviewer to explore the research frontiers and hotspots of CTO lesions which can provide reference for related researchers and clinicians.

## 2. Data and methods

### 2.1. Data sources

A search was conducted in the Web of science database from 1999 to April 2024 under the title “Chronic total occlusion.” A total of 4416 articles were retrieved, with 3971 articles remaining after removing peripheral vascular system papers, conference papers and retracted publications. The retrieval date was April 2024.

### 2.2. Data analysis

The Web of Science database literature analysis tool was utilized to count the yearly trend of publications. Cite Space 6.3.R1 and VOSviewer 1.6.18 software were utilized for country/region, research institution, author and keyword analysis respectively. The time span was set from 1999 to 2024, and the length of individual time partitions was set to 1 year.

## 3. Results

### 3.1. Search results

**Figure 1.**
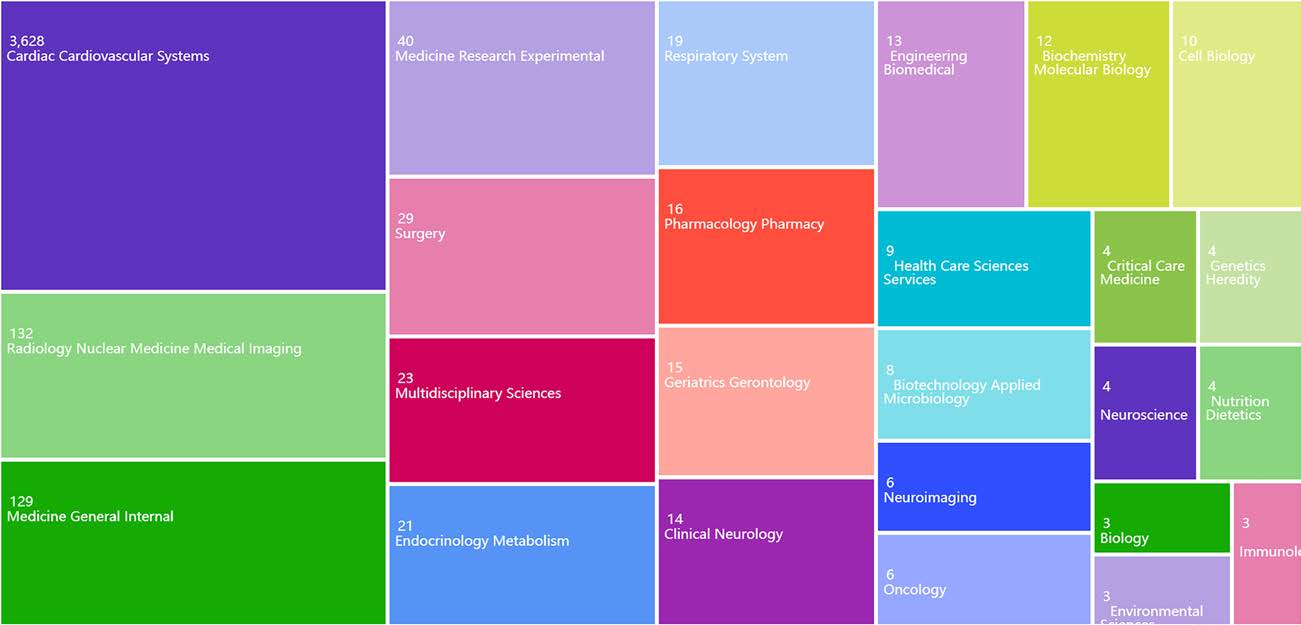
Literature categories in Web of science for coronary CTO. CTO = chronic total occlusion.

**Figure 2.**
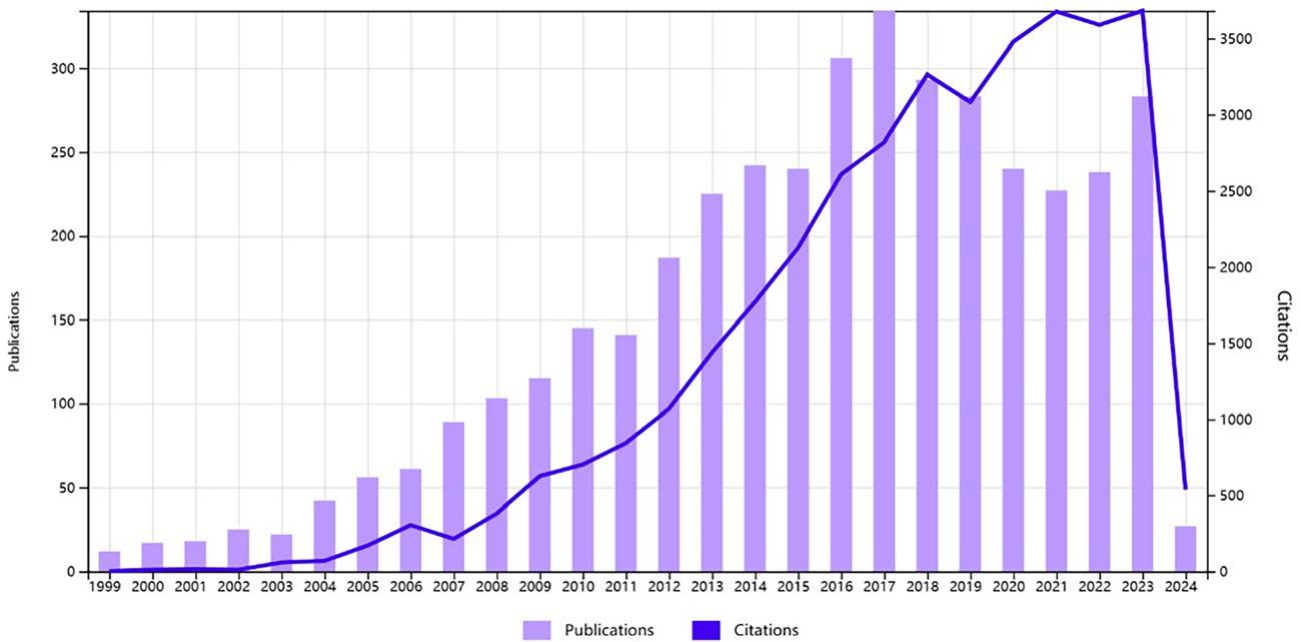
Publication and citation list of coronary CTO from 1999 to 2024. CTO = chronic total occlusion.

As shown in Figure 1, the search results were analyzed by Web of Science, and it was found that the literature categories were mainly concentrated in Cardiac Cardiovascular Systems and Radiology Nuclear Medical Imaging, and there were relatively few publications in other literature categories. As shown in Figure 2, the peak of the number of publications in this field is mainly concentrated in 2016 to 2019, with the annual number of publications above 250, of which the highest number of publications was 334 in 2017. The number of articles in recent years is in a stable stage, but in 2021 the number of articles starts to show an increasing trend again. The citation rate of articles in the field of this disease continues to rise, indicating that there are still many research hotspots for this disease, and there are still many researchers actively exploring in this field.

### 3.2. National/regional distribution and cooperation network

**Figure 3.**
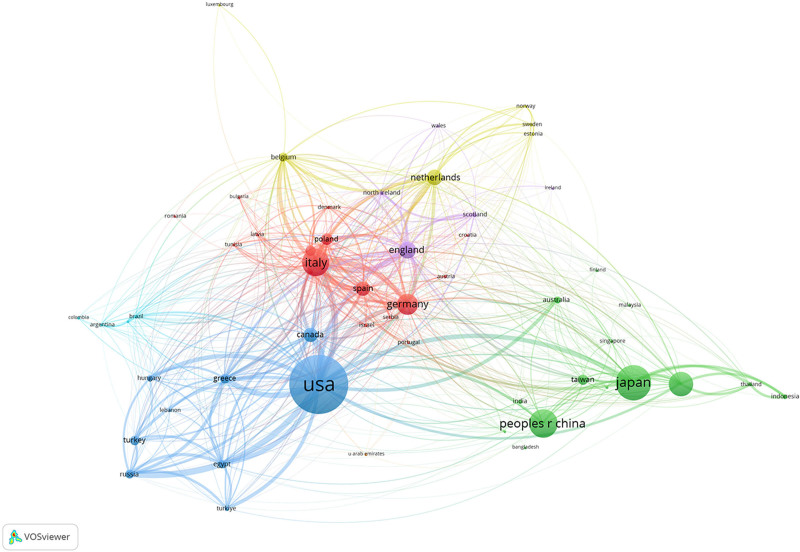
Country linkages analyzed by VOSviewer regarding coronary CTO research. CTO = chronic total occlusion.

**Figure 4.**
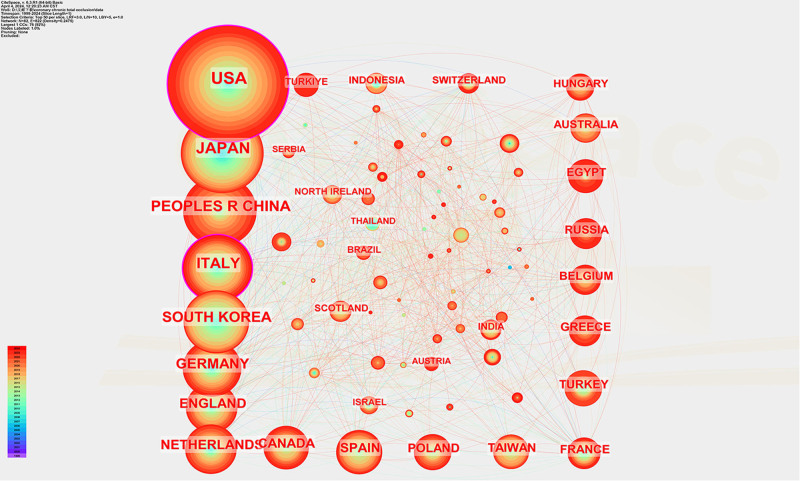
Relevant countries for coronary CTO research. CTO = chronic total occlusion.

Importing the data from Web of Science into Citespace for analysis, a total of 82 countries around the world have published articles in this field from 1999 to 2024. As shown in Table 1, the country with the highest number of articles published in this field is USA (n = 1093), followed by Japan (n = 577), People’s Republic of China (n = 449), Italy (n = 415), South Korea (n = 362), Germany (n = 303), England (n = 242), the Netherlands (n = 218), Canada (n = 187), and Spain (n = 186). Of all the countries involved, USA and Japan published >40% of the total number of articles. The network linkages analyzed by Citespace and VOSviewer show that in terms of article linkages, the USA and Japan have more linkages with countries around the world. It indicates that the United States and Japan are more authoritative in the research of chronic complete coronary artery disease during the period. China, on the other hand, has fewer line contacts with other countries, although it ranks third in the number of published articles in this field. It indicates that China’s research in coronary CTO is relatively independent and has relatively few contacts with other countries. As shown in Figure 3, the color of China is mainly green, indicating that the relevant articles published in China are mainly concentrated around 2018. After visualization and analysis, as shown in Figure 4, the proportion of the orange–red range is higher, indicating that the research on coronary CTO is mainly concentrated after 2017.

**Table 1:** Top 10 countries for the number of publications on coronary chronic total occlusion.

Rank	Countries	Articles counts	Percentage
1	USA	1093	27.52%
2	Japan	577	14.53%
3	People’s R China	449	11.31%
4	Italy	415	10.45%
5	South Korea	362	9.12%
6	Germany	303	7.63%
7	England	242	6.09%
8	Netherlands	218	5.49%
9	Canada	187	4.71%
10	Spain	186	4.68%

### 3.3. Distribution of institutions and number of publications

**Figure 5.**
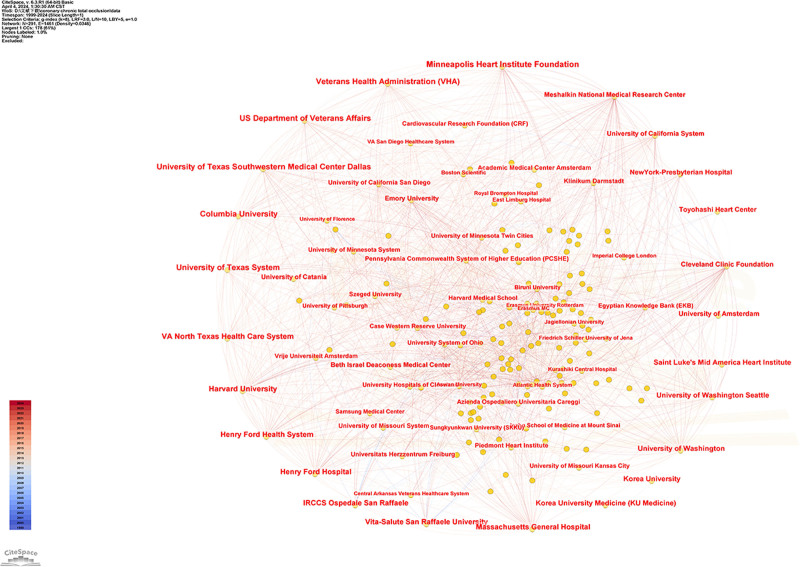
Institutions associated with coronary CTO research. CTO = chronic total occlusion.

The analysis revealed that a total of 291 research organizations have published articles related to coronary CTO. As shown in Table 2, the top 10 research organizations in terms of the number of articles published were Minneapolis Heart Institute Foundation, US Department of Veterans Affairs, Veterans Health Administration, University of Texas System, Columbia University, University of Texas Southwestern Medical Center Dallas, VA North Texas Health Care System, Harvard University, Henry Ford Health System, and Henry Ford Hospital. The top 10 research institutions are all from the United States. As shown in Figure 5, each dot represents a research institution, and the connecting line represents the relevant cooperation between institutions, which shows that there is a close cooperation between the top 10 research institutions in terms of the number of published articles and each other institution.

**Table 2:** Top 10 institutions for the number of publications on coronary chronic total occlusion.

Rank	Institutions	Article counts	Percentage	Country of affiliation
1	Minneapolis Heart Institute Foundation	276	6.95%	USA
2	US Department of Veterans Affairs	244	6.14%	USA
3	Veterans Health Administration	244	6.14%	USA
4	University of Texas System	216	5.44%	USA
5	Columbia University	212	5.34%	USA
6	University of Texas Southwestern Medical Center Dallas	205	5.16%	USA
7	VA North Texas Health Care System	189	4.76%	USA
8	Harvard University	177	4.46%	USA
9	Henry Ford Health System	155	3.90%	USA
10	Henry Ford Hospital	155	3.90%	USA

### 3.4. Visualization analysis of authors of the coronary CTO study

**Figure 6.**
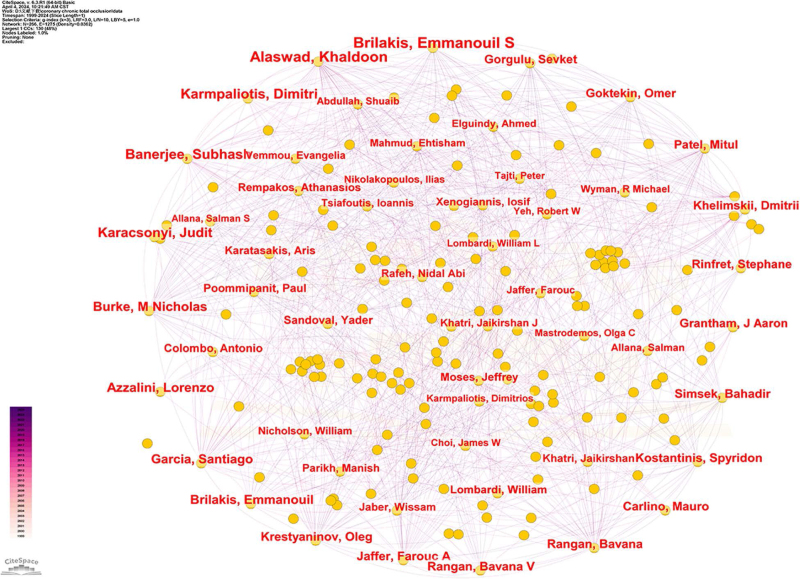
Relevant authors of coronary CTO studies. CTO = chronic total occlusion.

**Figure 7.**
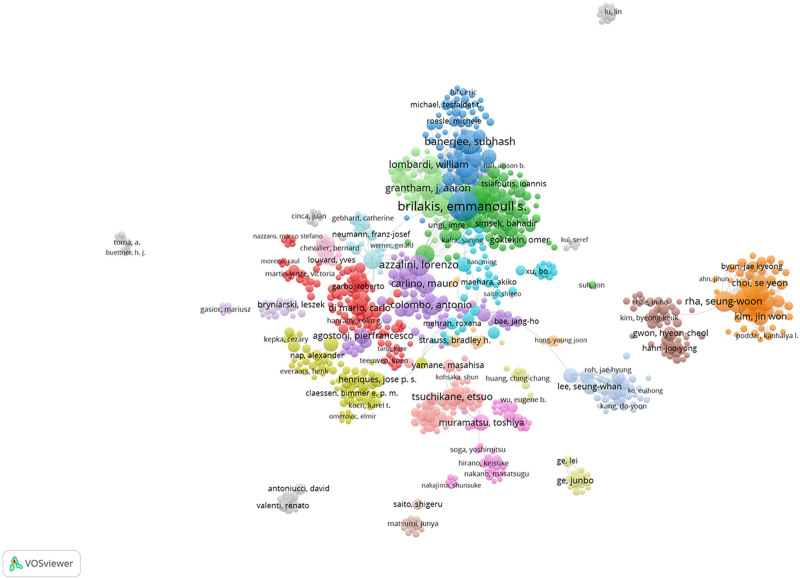
Relevant author collaborations analyzed by VOSviewer regarding coronary CTO research. CTO = chronic total occlusion.

Visual analysis revealed that a total of 266 authors published articles related to coronary CTO, as shown in Table 3 and Figure 6, with the top 10 authors publishing a total of 1506 related articles. Brilakis, Emmanouil S, University of Texas Southwestern Medical Center, USA, published the highest number of articles, totaling 280. The second-highest number of articles was published by Alaswad, Khaldoon, Henry Ford Hospital, USA, with 219 articles. The 2 authors’ areas of research focused on the pathogenesis, treatment, and adverse cardiovascular outcomes of CTO in relation to chronic totally occlusive coronary artery disease. They found that chronic totally occlusive coronary artery disease was able to lead to a significant increase in mortality and increased incidence of adverse cardiovascular events in patients. Cluster analysis of the authors revealed that studies on CTO focused on 5 main areas: #0 progress-CTO registry; #1 contemporary multicenter registry; #2 long-term outcome; #3 percutaneous recanalization; and #4 periprocedural myocardial injury. As shown in Figure 7, based on the visual analysis of the authors, we can get a clear picture of the world’s leading scholars on coronary CTO research. They clustered in different research points to develop close collaboration. By searching the relevant articles of authoritative authors, we can understand their therapeutic ideas and innovative ideas in this disease and grasp the current research hotspots and frontiers, which helps to save research time and improve research efficiency.

**Table 3:** Authors of the top 10 published articles on coronary chronic total occlusion.

Rank	Authors	Article counts	Article percentage
1	Brilakis, Emmanouil S	280	7.05%
2	Alaswad, Khaldoon	219	5.51%
3	Karmpaliotis, Dimitri	162	4.08%
4	Banerjee, Subhash	160	4.03%
5	Karacsonyi, Judit	144	3.63%
6	Burke, M Nicholas	122	3.07%
7	Azzalini, Lorenzo	120	3.02%
8	Garcia, Santiago	106	2.67%
9	Brilakis, Emmanouil	103	2.59%
10	Krestyaninov, Oleg	90	2.27%

### 3.5. Visual analysis of keywords

#### 3.5.1. Keyword analysis

**Figure 8.**
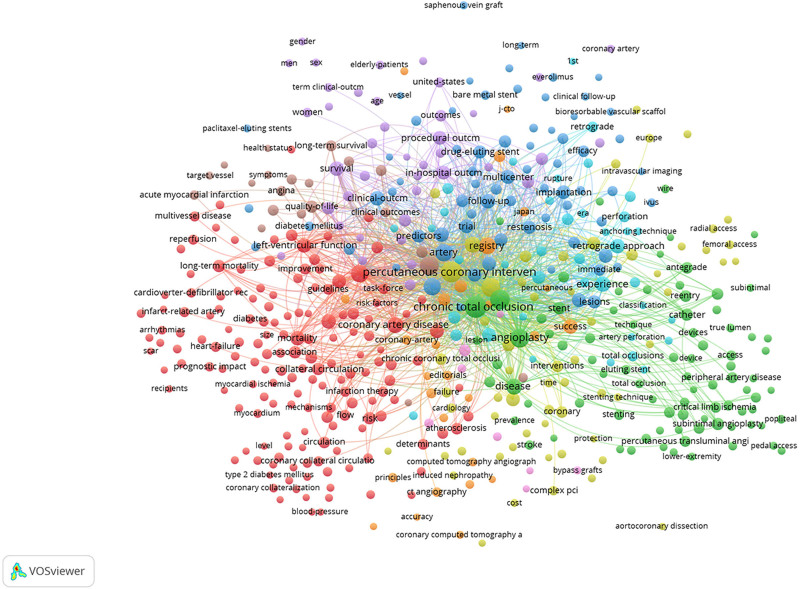
Keyword analysis of articles related to coronary CTO. CTO = chronic total occlusion.

As shown in Figure 8, the nodes in the co-occurrence network graph indicate the corresponding keywords, the size of the nodes indicates the number of articles containing the keywords, and the connecting lines between the nodes indicate the relationship between the keywords. Visual analysis of all the keywords of the retrieved articles revealed a total of 289 important keywords were retrieved, and the connecting lines between the keywords totaled 2848, of which 26 keywords appeared >100 times in frequency. As shown in Table 4, the top 20 keywords in terms of frequency of occurrence, among which the top 10 keywords and their frequency are as follows: CTO (912); PCI (867); recanalization (530); revascularization (435); outcome (421); artery (347); intervention (325); impact (328); angioplasty (327); registry (285). The most frequent keyword was CTO (912 times), followed by PCI (867 times) and recanalization (530 times). This shows that research on CTO has focused on the study of the disease as well as PCI treatment and revascularization after total coronary occlusion. The top 10 high center keywords were: angioplasty (0.14); artery (0.10); recanalization (0.08); intervention (0.08); coronary occlusion (0.08); balloon angioplasty (0.07); disease (0.06); success (0.06); clinical outcome (0.06); angiography (0.06); revascularization (0.05); outcome (0.05); artery disease (0.05); in hospital outcome (0.05); and implantation (0.05).

**Table 4:** Top 20 keywords in terms of frequency and centrality of coronary chronic total occlusion.

Rank	Key words	Frequency	Average age	Centrality
1	Chronic total occlusion	912	2003	0.03
2	Percutaneous coronary intervention	867	2006	0.03
3	Recanalization	530	1999	0.08
4	Revascularization	435	2004	0.05
5	Outcome	421	2005	0.05
6	Artery	347	1999	0.10
7	Intervention	335	2004	0.08
8	Impact	328	2009	0.04
9	Angioplasty	327	1999	0.14
10	Registry	285	2011	0.02
11	Insights	203	2011	0.04
12	Coronary artery disease	187	2002	0.04
13	Experience	159	2004	0.02
14	Disease	149	2002	0.06
15	Left ventricular function	139	2006	0.04
16	Success	138	1999	0.06
17	Metaanalysis	132	2011	0.02
18	Clinical outcome	126	2011	0.06
19	Artery disease	126	1999	0.05
20	Retrograde approach	115	2008	0.02

#### 3.5.2. Cluster analysis of keywords

**Figure 9.**
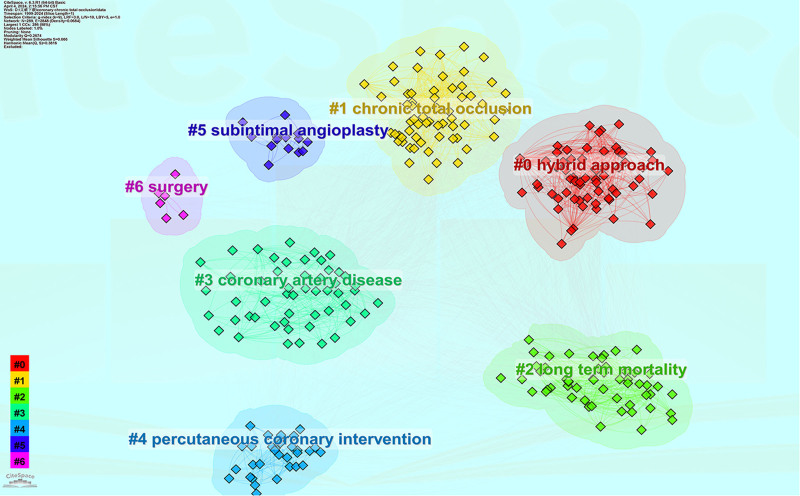
Keyword clustering analysis.

The co-occurring keywords were analyzed by clustering to reveal the main themes of the keywords in the relevant categories. As shown in Figure 9, in the study, we performed cluster analysis of the keywords and found a total of 7 clusters, which are: #0 hybrid approach; #1 CTO; #2 long term mortality; #3 coronary artery disease; #4 PCI; #5 subintimal angioplasty; and #6 surgery. As shown by the visual analysis, studies on CTO coronary artery disease have focused on mixed-modality treatment, PCI treatment, surgical treatment, long-term adverse cardiovascular outcome events, and the impact on patients’ later lives.

#### 3.5.3. Burst word analysis

**Figure 10.**
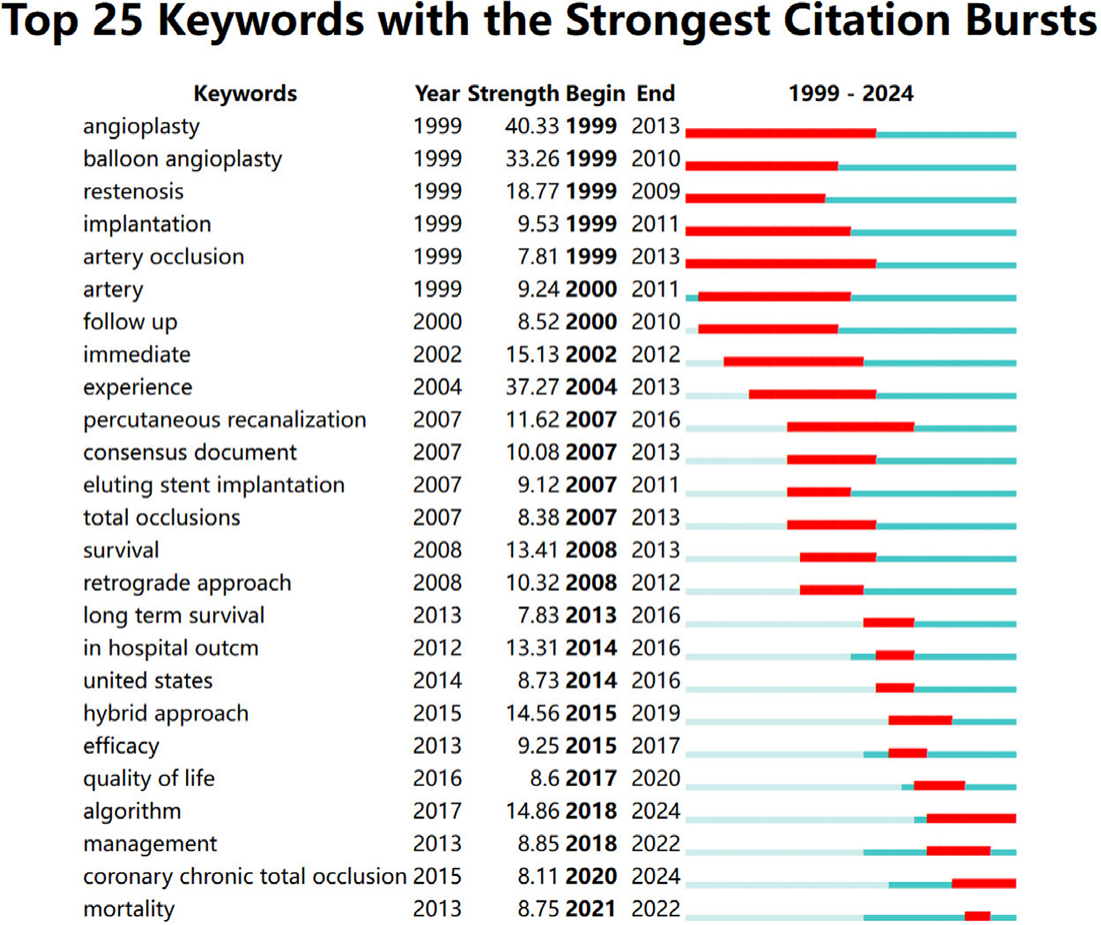
Top 25 outbreak keywords.

An outbreak word indicates a keyword that is frequently cited during a specific time period. As shown in Figure 10, the top 25 cited eruption words are: angioplasty; balloon angioplasty; restenosis; implantation; artery occlusion; artery; follow up; immediate; experience; percutaneous recanalization; consensus document; eluting stent implantation; total occlusion; survival; prograde approach; long term outcome; united states; hybrid approach; efficacy; quality of life; algorithm; management; coronary CTO; mortality. Where the blue line represents the entire time period from 1999 to 2024, the red line represents the time period in which the keyword citation broke out, indicating that the keyword was cited more frequently in that time period. From Figure 10, it can be seen that coronary artery occlusion and angioplasty were more explosive and more researched within the academic community until 2013. Over time, studies related to retrograde access to open vessels and patient survival became the main hotspot in 2008 to 2012. Studies related to hybrid access to open vessels and long-term survival emerged again in 2015 to 2019. The outbreak keywords in the last 5 years were: quality of life, algorithm, management, coronary CTO, and mortality. Algorithmic scoring systems, management, and mortality have become relevant hotspots in CTO research.

#### 3.5.4. Visual analysis of keywords using time axis

**Figure 11.**
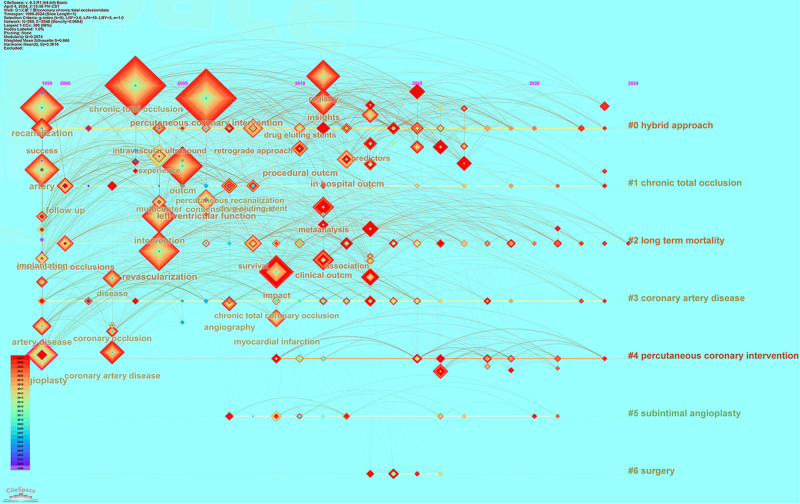
Keyword timeline graph for coronary CTO. CTO = chronic total occlusion.

The timeline graph can reflect the historical evolution and frontiers of hotspots in the research field in recent years. The keyword clustering of studies has continued until now, mainly involving: #0 hybrid approach; #1 CTO; #2 long term mortality; #3 coronary artery disease; #4 PCI; #5 subintimal angioplasty; and #6 surgery. As shown in Figure 11, analysis of the Citespace timeline shows that studies prior to 2010 focused on PCI, blood reperfusion, and interventional therapies, with the mean number of years of study concentrated around 2005. Studies after 2010 focused on myocardial infarction after CTO of coronary arteries, prediction of clinical outcomes, and pharmacologic balloon and hybrid approaches for the treatment of occluded coronary arteries. For hybrid access for the treatment of CTO of coronary arteries, studies between 2005 and 2015 were more focused and formed correlation research between pre and posttreatment. A full range of studies have also been conducted on pretreatment success rate calculation models, difficulty prediction and analysis, and posttreatment management.

## 4. Discussion

### 4.1. Countries and institutions

Currently, coronary CTO research countries and research organizations are mainly led by the United States. In terms of the number of published articles, the US leads, followed by Japan and China. All of the top 10 research organizations in the world are from the United States. Thus, the United States is at the forefront of coronary CTO research and deserves to be studied and emulated by other countries.^[21]^ Although China and Japan are at the forefront of procedures for the treatment of this disease, mainly based on a large population base, the improvement of living standards results in a large number of patients, but there are fewer authoritative studies and innovative discoveries. Therefore, China and Japan, as latecomers, should grasp the research trend of this disease, look up to the former, and strengthen cooperation to overcome the difficulties in the treatment of this disease and better maintain human health.

### 4.2. Authors

Visual analysis shows that the most influential author in coronary CTO research is Brilakis, Emmanouil S from the United States, with a total of 280 publications.^[22,23]^ The most frequently cited article was “Prediction of guidewire successful traversal of natural coronary lesions with CTO within 30 minutes: the Japan-CTO score as a difficulty grading and time assessment tool” by Yoshihiro Morino from Japan.^[24]^ This article is innovative in presenting a simple and practical algorithm for the difficulty of treating coronary CTO lesions. It opens a new research paradigm and enables the prediction of the opening or non-opening of occluded coronary arteries. The proposed model also leads the “Establishment of algorithmic systems for the opening and difficulty of chronic total occlusion of coronary arteries” to become one of the latest research hotspots. Visual analysis of the authors shows that the high-impact authors are mainly from the United States and Japan. CTO researchers from other countries can communicate more with American and Japanese researchers to learn their advanced treatment methods and concepts. At the same time, more in-depth research can be carried out on their basis.

### 4.3. Treatment, mortality, management, and algorithm system for chronic total coronary artery occlusion

#### 4.3.1. Therapeutic methods

There are many treatment methods regarding CTO of coronary arteries. Currently, the main methods are: (1) Orthogonal guide antegrade wire escalation: The antegrade wire escalation technique is the most commonly used technique for CTO lesions to pass the guide wire through the occluded lesion during PCI surgery, with a utilization rate of 66% to 78%, especially more suitable for the application of those who have milder CTO lesions.^[25–27]^ (2) Antegrade dissection reentry: It was first reported by Colombo et al,^[28]^ but this technique is prone to cause adverse cardiovascular events such as subendocardial hematoma and myocardial infarction. (3) Retrograde wire escalation and kissing wire technique: It is more practical for opening complex occluded coronary arteries, which is mastered by most of the skilled CTO operators and has a high success rate of opening.^[29,30]^ (4) Reverse subendocardial reentry: For CTO lesions with long occluded coronary arteries, severe calcification, and difficulty in passing the guidewire with multiple attempts, the application of this technique can improve the passage rate of the lesion, and the success rate is increasing year by year.^[31]^ (5) Hybrid Surgical Strategy: This technique was first proposed by Brilakis et al. The hybrid approach requires a high degree of operator proficiency, and the use of this technique has increased the success rate of PCI for CTO to about 90%.^[32,33]^ The generation of various methods of opening occluded coronary arteries today has led to a rapid development of interventional therapy for CTO of coronary arteries and has enabled many patients to benefit from the development of this technology. Interventional methods for CTO lesions have been a major focus of research in recent years, and the maturity of the techniques continues to increase. However, there is still a lack of systematic research on the comparative analysis of the advantages and disadvantages, success rate and safety of each treatment technique. This warrants further in-depth study to better master advanced treatment methods and provide the best treatment strategy for patients.

#### 4.3.2. Management and mortality

With clinical treatment, it has been found that the need for vessel opening in patients with CTO of coronary arteries is equally a question worth exploring. Successful opening of CTO vessels can restore blood supply, improve patients’ ischemic symptoms, and restore cardiac function. However, not all patients with CTO can benefit after opening the occluded vessel. In some patients, the occurrence of coronary occlusion has resulted in necrosis of cardiomyocytes, which is not satisfactory in terms of symptom improvement and recovery of cardiac function in patients after vessel opening.^[34]^ Visual analysis by Citespace and VOSviewer shows that nowadays, the mortality and survival management of patients with CTO of coronary arteries is also an emerging hot topic of research in recent years. This makes us realize that cardiovascular interventionalists cannot only focus on the opening of occluded vessels. The clinical outcome and prognosis of the patients after the opening of the vessels are also our concern and deserve our in-depth research and analysis.

Coronary CTO lesions are difficult to treat because of the long duration of occlusion and calcification, and they are a procedure with a high coefficient of difficulty within coronary interventions.^[35]^ Currently, 50% of the patients with coronary CTO receive drug therapy. And only 20% receive coronary intervention to open the occluded coronary artery, and the success rate is around 70%.^[36]^ With the generation of new techniques and devices in recent years, the success rate of opening coronary CTO lesions in certain skilled centers is now around 90%.^[37]^ In the clinic, for acute totally occluded lesions, the vessel should be opened quickly to restore blood supply and save a large number of dying cardiomyocytes.^[38]^ For CTO lesions, the coronary arteries have more branch vessels. When a slow occlusion occurs in 1 coronary artery branch, it triggers other branch vessels to form collateral circulation to supply the corresponding ischemic site, and there is no obvious myocardial ischemia manifestation. Whether such patients need to receive treatment should be evaluated and analyzed according to the actual situation of the patients.^[39]^ Clinical treatment has revealed that certain patients with open coronary CTO did not benefit much, and the restoration of blood supply to the occluded coronary artery still failed to rescue necrotic cardiomyocytes. Consequently, this has led clinicians and researchers to question the necessity of treating CTO lesions and to begin studies related to the survival management of CTO patients. According to the visualization and analysis of Citespace and VOSviewer, it can be seen that studies in recent years have focused on patient mortality, adverse outcomes, and patient survival management. The impact of the opening of CTO coronary arteries on patient mortality and quality of life is being actively studied.

#### 4.3.3. Algorithm system

In the past, research has focused on innovations in techniques, surgical therapies, and preoperative and postoperative treatment of patients, and the current understanding of the disease is becoming more comprehensive. Today’s research focuses on the overall evaluation of the patient’s coronary vasculature and emphasizes the importance of preoperative evaluation. Preoperative systematic evaluation of the surviving myocardium and the difficulty of surgery is performed before considering surgical treatment, rather than blindly performing coronary CTO surgical treatment.^[40–43]^ Many scoring systems on the success rate and surgical difficulty of PCI surgery for CTO are available today, mainly: the CL (Clinical and lesion-related, CL) point system,^[44]^ the Ellis point system,^[45]^ the Japan-CTO score,^[24]^ the ORA (Ostial, Rentrop grade, Age) point system,^[46]^ PROGRESS-CTO point system,^[33]^ Antegrade CTO point system,^[47]^ and RECHARGE point system.^[48]^ Therefore, one of the current research hotspots is the study of algorithmic systems for the difficulty of coronary CTO treatment, by which the success rate of coronary CTO opening can be evaluated. These integral systems for evaluation have their own guiding value for opening different types of coronary CTO lesions. Cardiovascular interventionalists can assess the probability of success in opening occluded lesions by using the point system and also can know the success rate of the procedure in the preoperative period. Patients with a low probability of success can be informed in advance, avoiding the pain and expense associated with surgical attempts.

## 5. Future prospects and clinical significance

The visual analysis of bibliometrics shows that the current research hotspots of coronary CTO lesions mainly focus on the innovation of treatment methods, patient mortality, long-term prognosis and algorithmic assessment of the difficulty of opening CTO lesions. With the continuous emergence of new technologies and concepts, the interventional treatment of coronary CTO lesions will become more mature and widespread, and the efficacy and safety of CTO recanalization will be further improved. With advanced treatment concepts, appropriate treatment strategies and procedures can be selected for CTO patients. The risk of complications of CTO recanalization will be reduced, the safety of the procedure will be improved, and the occurrence of adverse cardiovascular events will be reduced. The application of new technologies such as drug-eluting stents, drug balloons and intravascular ultrasound will reduce the rate of postoperative restenosis and improve the long-term prognosis of patients after CTO recanalization.^[49,50]^ The impact of CTO recanalization on patients’ prognosis is clarified by large-sample, long-term follow-up studies comparing the differences in endpoint events between treated and non-treated patients, which provide a basis for clinical decision-making. Preoperative assessment methods are used to help identify patients who are most suitable to undergo CTO recanalization so that patients can avoid unnecessary treatment and reduce their financial burden.^[51]^ Through the analysis and research in this article, cardiovascular interventionalists and clinicians can grasp the latest treatments and therapeutic strategies for the disease and provide more optimal treatment plans for patients with coronary CTO. Coronary CTO researchers can summarize the latest hotspots and frontiers of the disease research, which helps to grasp the latest research trends of the disease. This will benefit more CTO patients.

## 6. Conclusion

In this paper, the literature related to coronary CTO was visualized and analyzed by Citespace and VOSviewer. The number of articles on coronary CTO lesions has been generally increasing year by year, and the number of publications has stabilized in recent years. Research institutions and authors in the United States lead the world in terms of the number of articles published and their impact. We recommend more active collaboration between countries, institutions and authors for clinical research and technological innovation. Ongoing research is focused on treatments for CTO of coronary arteries, algorithmic assessment of the difficulty of surgical treatment, clinical outcomes and management of patients. With the innovative development of modern technologies and the increasing level of treatment, the number of procedures for CTO treatment has increased, and the therapeutic strategy for the opening of CTO lesions has been transformed. Research in CTO lesions is no longer focused solely on innovations in therapeutic approaches. Algorithmic assessment systems, clinical outcome management of patients treated or not treated, and theoretical innovations in coronary intervention for chronic total occlusive disease are also concerns in clinical care. This clarifies the hotspots and directions of research for cardiovascular clinicians and coronary CTO investigators. With technological innovations and research advances, interventionalists, noninterventionalists, rehabilitation physicians and geriatricians in cardiovascular medicine can collaborate to provide more refined management of CTO patients based on the needs of the patient’s condition.

## 7. Strengths and limitations

This is a bibliometric analysis study using Citespace and VOSviewer. The visualization from hotspots and frontiers shows the progress of research on CTO of coronary arteries and the collaboration between countries, institutions and authors. However, some limitations remain. We only analyzed relevant studies from Web of Science, and the data may not be comprehensive enough. In addition, because of the existence of multiple synonyms, there may be some overlap between various content categories when clustering keywords.

## Author contributions

**Conceptualization:** Shudi Li, Wenwen Li.

**Data curation:** Shudi Li, Yunxiao Zhang.

**Formal analysis:** Menghe Zhang, Wenwen Li, Zhenhai Sun, Yunxiao Zhang, Yaoyao Zuo, Shouqiang Chen.

**Funding acquisition:** Yaoyao Zuo, Shouqiang Chen.

**Investigation:** Menghe Zhang, Wenwen Li.

**Methodology:** Shudi Li, Yunxiao Zhang.

**Project administration:** Menghe Zhang, Zhenhai Sun, Yaoyao Zuo.

**Resources:** Zhenhai Sun, Yaoyao Zuo.

**Software:** Shudi Li, Zhenhai Sun, Yaoyao Zuo.

**Supervision:** Menghe Zhang, Yunxiao Zhang, Shouqiang Chen.

**Validation:** Yunxiao Zhang, Shouqiang Chen.

**Visualization:** Shudi Li, Wenwen Li, Zhenhai Sun.

**Writing – original draft:** Shudi Li, Wenwen Li.

**Writing – review & editing:** Menghe Zhang, Shouqiang Chen.
